# Supplementary material for: NMR-based quantification of liquid products in CO2 electroreduction on phosphate-derived nickel catalysts
Source: Commun Chem. 2023 Jul 10;6:147. doi: 10.1038/s42004-023-00948-9 (PMC10333308; doi:10.1038/s42004-023-00948-9)
Supplement: Supplementary file 2 — Supplementary Information [file 42004_2023_948_MOESM2_ESM.pdf]

Supplementary Information

NMR-based quantification of liquid products in CO_2 electroreduction formed on phosphate-derived nickel catalysts

Phil Preikschas, Antonio J. Martín, Boon Siang Yeo, and Javier Pérez-Ramírez

Table of Contents

Supplementary Notes 2
Supplementary Discussion 4
Supplementary Tables 5
Supplementary Figures 8
Supplementary References 27

Supplementary Notes

Supplementary Note 1 | Details on error calculation

Errors in the determination of Faradaic efficiencies FEs were estimated based on a prospective quantification error of 5 ppm in product quantification. Concentrations of products c_i were calculated for eCO_2RR at a current density i of 10 mA cm^{-2} and a mass flow of 10 mLn min^{-1} according to an Anderson-Schulz-Flory distribution with an α-value of 0.4. A methane concentration of 250 ppm was assumed. The instrumental error was propagated through equation (**1**) with a Taylor series approximation resulting in electron-number-dependent uncertainties.

$$\mathrm{FE}_j = \frac{\dot{n} c_j z F}{i} \quad (\mathbf{1})$$

Where FE_j is the Faradaic efficiency of a specific product j, $\dot{n}$ is the molar flow, z is the number of transferred electrons, and F is the Faraday constant. Relative errors were calculated from the resulting uncertainties.

Supplementary Note 2 | Details on the Automated Product Analysis Routine (APAR)

APAR is written in Python and is designed to automate the determination of concentrations and Faradaic efficiencies of eCO_2RR products from NMR raw data. This routine makes use of several open-source Python packages: (1) *Nmrglue*[3] for reading NMR raw data and processing, (2) *NumPy*[4] and (3) *SciPy*[5] for handling multidimensional arrays and mathematical operations, (4) *pandas*[6] for data export, (5) *matplotlib*[7] for data visualization, and (6) *lmfit*[8] for peak fitting and deconvolution. Raw data of different proprietary file formats can be used (*e.g.*, recorded on Bruker, Varian, and Agilent instruments). However, it is worth noting that the code was intensively tested with Bruker files. One or several NMR spectra can be evaluated using user-defined analysis schemes calling all required functions. An example analysis scheme is provided on GitHub (https://github.com/philpreikschas/apar) with the source code and detailed documentation of available functions. The main functions are related to six consecutive steps described briefly: (1) a series of pre-processing steps are performed (*e.g.*, apodization, zero-filling, phase correction, *etc.*) to increase the SNR, (2) chemical shift referencing based on detection of user-defined internal standard, (3) peak identification with adjustable threshold based on SNR, (4) product assignment using chemical shift positions and coupling constants reported in this study as **Supplementary Table 1**, (5) integration or fitting and deconvolution of identified peaks assigned to known eCO_2RR products, and (6) calculation of product concentrations based on an internal standard. The product assignment accounts for pH-dependent shifts once the sample's pH is set in the respective function. In the case basic electrocatalytic parameters are provided by the user, Faradaic efficiencies are determined.

Lastly, the results are exported in standard tabulated data formats or can be posted into a database for further data analysis.

Supplementary Note 3 | Calculation of standard reduction potentials

Standard reduction potentials were calculated using a similar approach reported by Nitopi and co-workers.[1] A MATLAB script was written for the calculation directly from tabulated thermodynamic data (**Supplementary Table 4**). The code is available on GitHub and can be used free of charge (https://github.com/philpreikschas/srp). Standard Gibbs free energies of formation were calculated *via* the Gibbs-Helmholtz equation. For liquid products, Gibbs free energies of solvation were determined based on Henry's law constants K_H taken from tabulated data[2] and then used for Gibbs free energies of formation for solvated compounds. K_H values of certain products were averaged from selected data providing the smallest standard deviations. Finally, standard reduction potentials were calculated based on the Nernst equation and reaction equations provided in **Table 1**.

Supplementary Note 4 | Determination of quantification limits

SNRs were calculated for all signals considered for quantification (**Table 2**) of reference samples containing common eCO_2RR products with concentrations ranging from 0.5 to 500 μM (**Supplementary Fig. 2**). For this purpose, the noise was determined from a signal-free region (7.00-8.00 ppm) as the standard deviation of all datapoints. SNRs were then derived as the ratio of signal intensity and noise value. For multiplets, the signal with the lowest intensity was considered. Quantification limits were then obtained by linear regression of the determined SNRs on the respective product concentrations, assuming an SNR of 6 as a threshold for reliable quantification (**Supplementary Fig. 6**). Quantification limits of other products (not included in reference samples) were estimated under the assumption that the sensitivity is independent of the analyte and were calculated from relative intensities obtained from the reference samples.

Supplementary Discussion

Combined approach for liquid product identification

Unknown compounds were identified based on a facile approach following three consecutive steps: (1) a selection of possible products was made based on chemical shift positions, multiplicities, and coupling constants from tabulated data[9], (2) ^{1}H NMR spectra of all possible compounds were predicted using *NMR Predict* package of the Mnova software suite to aid in the selection of appropriate reference samples, and (3) validation with selected reference samples showing the same signal pattern and a maximum deviation in chemical shift of 0.1 ppm between the reference and unknown compound. As Mnova only allows the prediction of NMR spectra in D_2O, electrolyte effects were compensated based on a set of ten reference samples (**Supplementary Fig. 4**). A systematic offset appeared in the corresponding parity plot of predicted and observed values. Thus, predicted values were corrected based on a linear regression resulting in an improved correlation between predicted and observed values (relative mean squared error was reduced from 10.6 to 6.67%).

Supplementary Tables

Supplementary Table 1 | Extended NMR parameters of observed products over PD-Ni catalysts and suitable internal standards.

CN[a]	Product	Probed nucleus	Chemical shift (ppm)			Multiplicity[b]	*J* coupling (Hz)	Electrons transferred[c]
			$KHCO_3$	KH_2PO_4	KOH			
1	Methanol	CH_3	3.23	3.23	3.21	s	-	6
	Formate	CHO^-	8.33	8.28	8.33	s	-	2
2	Ethanol	CH_3	1.06	1.05	1.05	t	6.2	12
		CH_2	3.53	3.52	3.52	q	7.2	
	Acetaldehyde	CH_3	1.20	1.20	1.20	d	5.2	10
	(diol)	CH	5.13	-[d]	-[d]	q	5.2	
	Acetaldehyde	CH_3	2.12	2.11	-[d]	d	3.0	10
		CH	9.55	9.54	-[d]	q	3.0	
	Acetate	CH_3	1.79	1.92	1.79	s	-	8
	Ethylene glycol	CH_2	3.54	3.54	3.54	s	-	10
3	*n*-Propanol	CH_3	0.77	0.75	0.76	t	7.5	18
		CH_2	1.42	1.41	1.41	sext	7.1	
		CH_2	3.44	3.43	3.42	t	6.7	
	i-Propanol	CH_3	1.05	1.04	1.04	d	6.2	18
		CH	3.90	3.89	3.89	hept	6.2	
	Propanal	CH_3	0.78	-[d]	-[d]	t	7.5	16
	(diol)	CH_2	1.48	-[d]	-[d]	m	-	
		CH	4.85	-[d]	-[d]	t	-[e]	
	Propanal	CH_3	0.92	-[d]	-[d]	t	7.3	16
		CH_2	2.44	-[d]	-[d]	qd	7.3, 1.4	
		CH	9.57	-[d]	-[d]	s	-	
	Hydroxyacetone	CH_3	2.02	-[d]	-[d]	s	-	14
		CH_2	4.25	-[d]	-[d]	s	-	
	Acetone	CH_3	2.10	2.10	2.10	s	-	16
	Allyl alcohol	CH_2	3.99	-[d]	-[d]	dt	5.3, 1,6	16
		CH_2	5.07	-[d]	-[d]	d	-	
		CH	5.89	-[d]	-[d]	m	-	
4	*n*-Butanal	CH_3	0.97	-[d]	-[d]	t	7.8	22
		CH_2	1.52	-[d]	-[d]	m	-	
		CH_2	2.28	-[d]	-[d]	m	-	
		CH	9.66	-[d]	-[d]	s	-	
	n-Butanol	CH_3	0.94	-[d]	-[d]	t	7.3	24
		CH_2	1.24	-[d]	-[d]	m	-	
		CH_2	1.42	-[d]	-[d]	m	-	
		CH_2	3.62	-[d]	-[d]	q	6.6	

[a] *CN*: carbon number.
[b] Signal multiplicities are abbreviated as follows: s: singlet, d: doublet, t: triplet, dt: doublet of triplets, q: quartet, sext: sextet, hept: heptet, m: multiplet.
[c] Number of electrons needed to form 1 mol of a specific compound from CO_2.
[d] Not detected during eCO_2RR experiments.
[e] Not detected due to overlap with water signal.

Supplementary Table 2 | Faradaic efficiencies for gaseous products from the catalytic evaluation of PD-Ni catalyst under different conditions.

#	Electrolyte	Potential (V vs. RHE)	Current density (mA cm^{-2})	FE (%)										
				H_2	CH_4	CO	C_2H_4	C_2H_6	C_3H_8	C_3H_6	C_4H_{10}	C_4H_8	C_5H_{12}	C_5H_{10}
1	1.0 M $KHCO_3$	−0.79	−6.12±0.95	64.6	0.14±0.00	3.10±0.26	0.54±0.01	0.52±0.01	0.64±0.01	0.38±0.33	tr[b]	tr[b]	N.D.[c]	N.D.[c]
2		−0.90	−12.6±4.6	82.9	0.33±0.03	0.93±0.16	0.29±0.03	0.33±0.03	0.34±0.05	0.30±0.04	0.37±0.06	tr[b]	N.D.[c]	N.D.[c]
3		−0.95	−47.5±0.3	80.0	0.67±0.02	1.62±0.02	0.16±0.00	0.17±0.00	0.16±0.00	0.12±0.00	0.14±0.00	0.12±0.00	0.14±0.01	0.13±0.01
4		−1.08	−109±5	79.5	1.34±0.04	1.29±0.05	0.22±0.02	0.19±0.02	0.16±0.01	0.09±0.01	0.09±0.01	0.07±0.00	0.07±0.01	0.06±0.00
5	0.1 M $KHCO_3$	−0.96	−10.7±0.4	68.3	1.86±0.09	2.75±0.29	0.68±0.02	0.62±0.01	0.68±0.02	0.49±0.01	0.61±0.01	0.49±0.01	0.58±0.00	0.54±0.00
6	0.5 M $KHCO_3$	−0.97	−22.4±2.1	80.7	1.18±0.11	1.85±0.17	0.34±0.01	0.36±0.01	0.29±0.01	0.31±0.01	0.26±0.03	0.23±0.02	0.29±0.03	0.27±0.02
7	1.0 M KH_2PO_4	−0.94	−70.6±1.6	94.0	0.07±0.04	0.01±0.01	0.06±0.01	0.06±0.00	0.05±0.04	0.04±0.04	tr[b]	tr[b]	N.D.[c]	N.D.[c]
8	1.0 M K_2CO_3	−0.94	−132±33	91.9	1.11±0.03	1.29±0.22	0.16±0.01	0.15±0.01	0.12±0.01	0.07±0.01	0.07±0.01	0.05±0.01	0.06±0.01	0.05±0.01
9	1.0 M KOH	−0.97	−175±20	82.6	1.47±0.19	0.80±0.01	0.22±0.01	0.20±0.02	0.09±0.01	0.14±0.00	0.05±0.01	0.06±0.00	0.05±0.01	0.04±0.00

[a] Summed Faradaic efficiencies (including liquid products) were above 80% except entry #1 (71%) due to the low current density resulting in low product concentrations.
[b] tr: trace amounts and below the quantification limit.
[c] N.D.: Not detected.

Supplementary Table 3 | Faradaic efficiencies for liquid products from the catalytic evaluation of PD-Ni catalyst under different conditions.

#	Electrolyte[a]	Potential (V vs. RHE)	Current denstiy (mA cm^{-2})	FE (%) (SNR[b])									
				Formate	Methanol	Acetate	Acet-aldehyde	Ethylene Glycol	Ethanol	Hydroxy-acetone	Acetone	*n*-Propanol	*i*-Propanol
1	1.0 M $KHCO_3$	−0.79	−6.12±0.95	0.95 (59)	0.27 (20)	0.15 (10)	N.D.[d]	N.D.[d]	0.34 (6)	N.D.[d]	tr[c]	N.D.[d]	N.D.[d]
2		−0.90	−12.6±4.6	0.39 (53)	0.14 (31)	0.05 (8)	N.D.[d]	0.07 (9)	0.33 (16)	N.D.[d]	0.04 (8)[e]	1.31 (50)[e]	0.22 (11)[e]
3		−0.95	−47.5±0.3	0.80 (168)	0.24 (113)	0.07 (16)	0.09 (8)[e]	0.02 (8)	0.24 (19)	0.03 (8)[f]	0.02 (10)[e]	0.12 (13)[e]	0.08 (11)[e]
4		−1.08	−109±5	0.69 (822)	0.15 (180)	0.01 (15)	0.03 (6)[e]	0.01 (8)	0.15 (29)	0.01 (8)[f]	0.01 (8)[e]	0.10 (29)[e]	0.05 (15)[e]
5	0.1 M $KHCO_3$	−0.96	−10.7±0.4	1.97 (435)[e]	0.64 (297)[e]	0.09 (30)[e]	0.18 (21)[e]	0.13 (24)[e]	0.54 (22)[e]	0.16 (24)[f]	0.11 (34)[e]	0.38 (21)[e]	0.40 (29)[e]
6	0.5 M $KHCO_3$	−0.97	−22.4±2.1	1.24 (327)	0.51 (174)	0.07 (17)	0.18 (8)[e]	0.06 (15)	0.44 (28)	0.08 (15)[f]	0.05 (8)[e]	0.28 (16)[e]	0.25 (21)[e]
7	1.0 M KH_2PO_4	−0.94	−70.6±1.6	0.03 (12)	0.02 (8)	0.07 (47)	N.D.[d]	tr[c]	0.15 (16)	N.D.[d]	tr[c]	0.31 (54)[e]	0.05 (9)[e]
8	1.0 M K_2CO_3	−0.94	−132±33	0.49 (551)	0.08 (114)	0.01 (20)	N.D.[d]	tr[c]	0.01 (14)	N.D.[d]	0.01 (8)[e]	0.07 (17)[e]	0.03 (10)[e]
9	1.0 M KOH	−0.97	−175±20	0.75 (925)	0.13 (142)	0.04 (44)	tr[c]	N.D.[d]	0.14 (22)	N.D.[d]	0.01 (7)[e]	0.08 (30)[e]	0.04 (23)[e]

[a] Representative ^{1}H NMR spectra for each electrolyte used are provided as **Supplementary Fig. 18**.
[b] SNR: signal-to-noise ratio.
[c] tr: trace amounts and below the quantification limit (SNR < 6).
[d] N.D.: Not detected (SNR < 2).
[e] ^{1}H NMR data was acquired with a relaxation delay of 5 s. Relaxation effects were compensated using correction factors obtained from signal recovery investigations (**Supplementary Fig. 3**).
[f] ^{1}H NMR data was acquired with a relaxation delay of 5 s. Relaxation effects were not compensated due to the low stability of hydroxyacetone.

Supplementary Table 4 | Thermodynamic data used for calculation of standard reduction potentials.

CN^a	Product	$\Delta_f H^0$ (kJ mol^{-1})^b	$\Delta_f S^0$ (J K^{-1} mol^{-1})	K_H (bar L mol^{-1})^d	$\Delta_f G^0$ (kJ mol^{-1})
1	Carbon monoxide	−110.5	197.7^b		−137.2 (g)
	Formic acid	−378.7	248.7^c	$1.73 \cdot 10^{-4}$	−351.0 (g)
					−372.5 (aq)
	Formate	−425.6	92.0^b		−370.7 (aq)
	Methanol	−201	239.9^b	$4.99 \cdot 10^{-3}$	−162.3 (g)
					−175.5 (aq)
	Methane	−74.6	186.3^b		−50.5 (g)
2	Acetic acid	−432.2	283.5^b	$2.16 \cdot 10^{-4}$	−374.3 (g)
					−395.2 (aq)
	Acetate	−486.0	86.6^b		−388.8 (aq)
	Acetaldehyde	−166.2	263.8^b	$7.33 \cdot 10^{-2}$	−133.0 (g)
					−139.5 (aq)
	Ethylene glycol	−392.2	303.8^b	$1.39 \cdot 10^{-5}$	−301.4 (g)
					−329.1 (aq)
	Ethylene	52.4	219.3^b		68.3 (g)
	Ethanol	−234.8	281.6^b	$5.71 \cdot 10^{-3}$	−167.9 (g)
					−180.7 (aq)
	Ethane	−84	229.2^b		−32.1 (g)
3	Hydroxyacetone			$7.70 \cdot 10^{1}$	
	Propionaldehyde	−185.6	304.5^b	$8.59 \cdot 10^{-2}$	−123.9 (g)
					−130.0 (aq)
	Acetone	−217.1	295.3^b	$3.63 \cdot 10^{-2}$	−152.6 (g)
					−160.9 (aq)
	Allyl alcohol	−124.5		$3.82 \cdot 10^{-3}$	
	Propylene	20.41	266.6^c		62.8 (g)
	n-Propanol	−255.1	322.6^b	$7.36 \cdot 10^{-3}$	−159.8 (g)
					−172.0 (aq)
	Propane	−103.8	270.3^b		−23.5 (g)
4	1,3-Butadiene	162.3	293^c		198.5 (g)
	Butanal	−204.8	343.7^b	$1.21 \cdot 10^{-1}$	−114.1 (g)
					−119.4 (aq)
	Butene	0.1	305.6^c		71.5 (g)
	n-Butanol	−274.9	362.8^c	$9.13 \cdot 10^{-3}$	−151.0 (g)
					−162.6 (aq)
	Butane	−125.7	310.1^c		−16.6 (g)
5	Pentene	−21.1	345.8^c		79.0 (g)
	Pentane	−146.9	349^c		−8.8 (g)
6	*n*-Hexene	−166.9	384.6^c		−37.7 (g)

[a] *CN*: carbon number.
[b] Data taken from CRC Handbook of Chemistry and Physics.[10]
[c] Data taken from Lange's Handbook of Chemistry.[11]
[d] Data taken from Compilation of Henry's law constants.[2]

Supplementary Figures

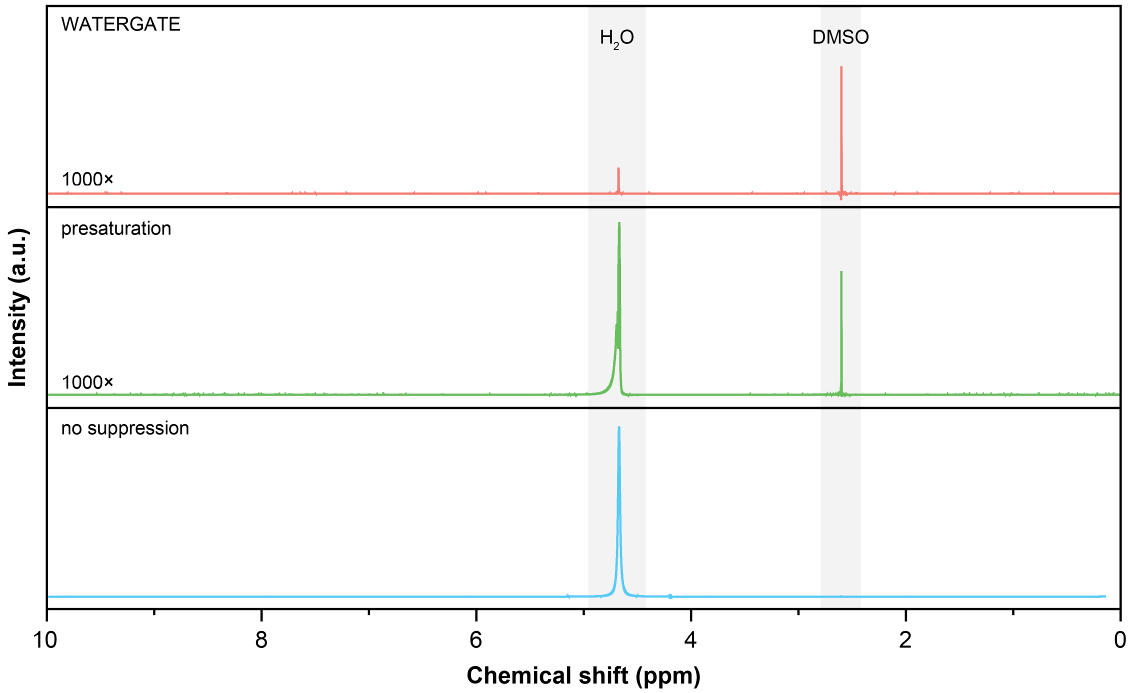

Supplementary Figure 1 | Comparison of water suppression methods on a reference sample. ^{1}H NMR spectra of a reference sample containing 3 mM DMSO with WATERGATE (red) and presaturation (green) for water suppression and without suppression (blue). Spectra were shift referenced and normalized on DMSO signal at 2.60 ppm. No further processing was applied.

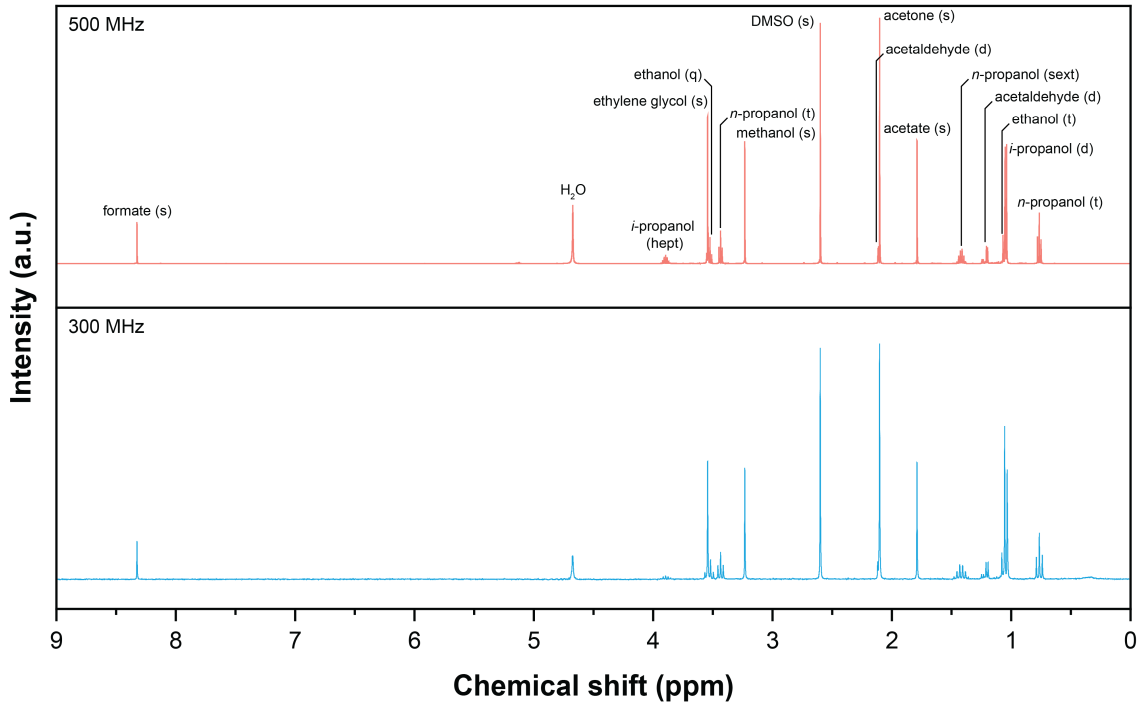

Supplementary Figure 2 | ^{1}H NMR spectra of reference sample. Reference sample containing 500 μM methanol, formate, ethanol, acetaldehyde, acetate, ethylene glycol, *n*-propanol, *i*-propanol, acetone, and DMSO as internal standard in CO_2-saturated 0.1 M $KHCO_3$ recorded on a 500 MHz (red) and 300 MHz spectrometer. Both spectra were recorded with a relaxation delay of 60 s (64 scans).

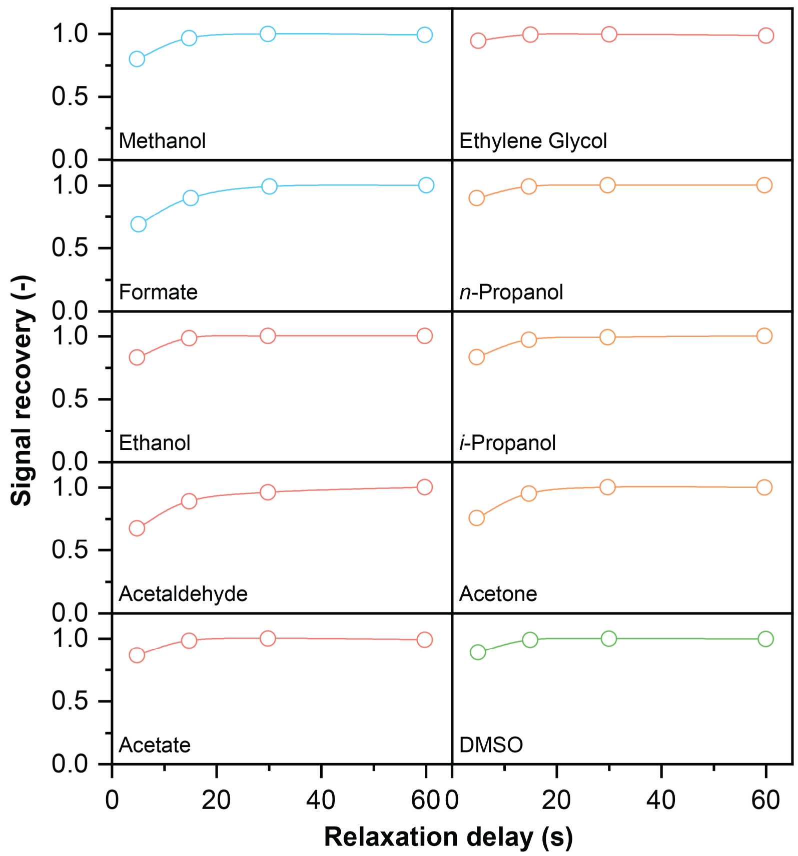

Supplementary Figure 3 | Signal recovery of common eCO_2RR products and DMSO as a function of relaxation delay. ^1H NMR spectra of the reference sample shown in **Supplementary Fig. 2** were recorded with varying relaxation delays (d1) of 5, 15, 30, and 60 s on a 500 MHz NMR spectrometer (64 scans). Signal recovery values were calculated from peak areas at the respective chemical shifts provided in **Table 2**.

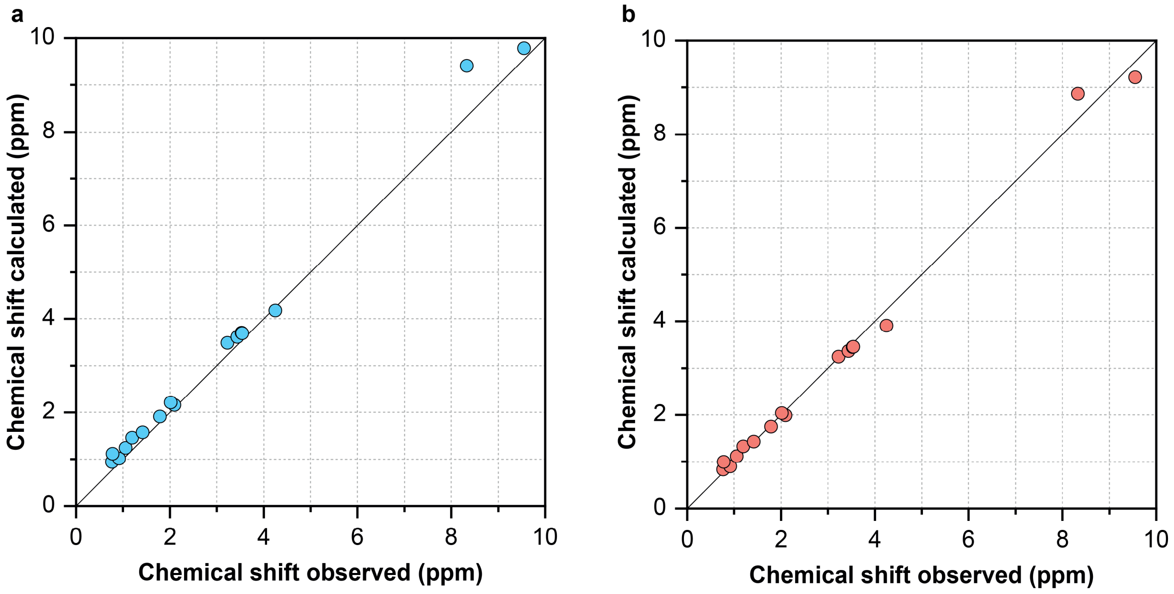

Supplementary Figure 4 | Chemical shift prediction of observed products over PD-Ni catalysts. Chemical shifts were predicted with the MNova software suite in D_2O. Parity plots of chemical shifts (**a**) before and (**b**) after offset-correction accounting for a 0.1 $KHCO_3$ electrolyte.

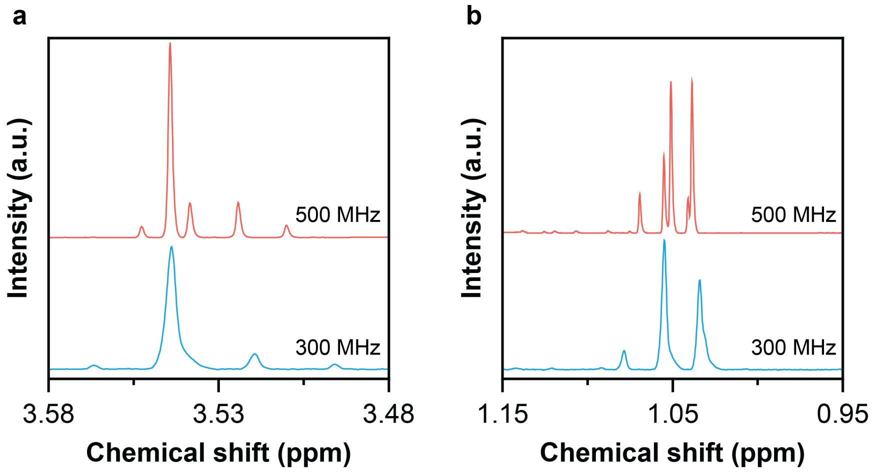

Supplementary Figure 5 | Comparison of ^{1}H NMR digital resolution. ^{1}H NMR spectra of the reference sample shown in **Supplementary Fig. 2** were recorded on a 500 MHz (red) and 300 MHz spectrometer (blue). Chemical shift region of (**a**) ethanol (q, 3.53 ppm) and ethylene glycol (s, 3.54 ppm) as well as (**b**) *i*-propanol (d, 1.04 ppm) and ethanol (t, 1.05 ppm). Both spectra were recorded with a relaxation delay of 60 s and 64 scans.

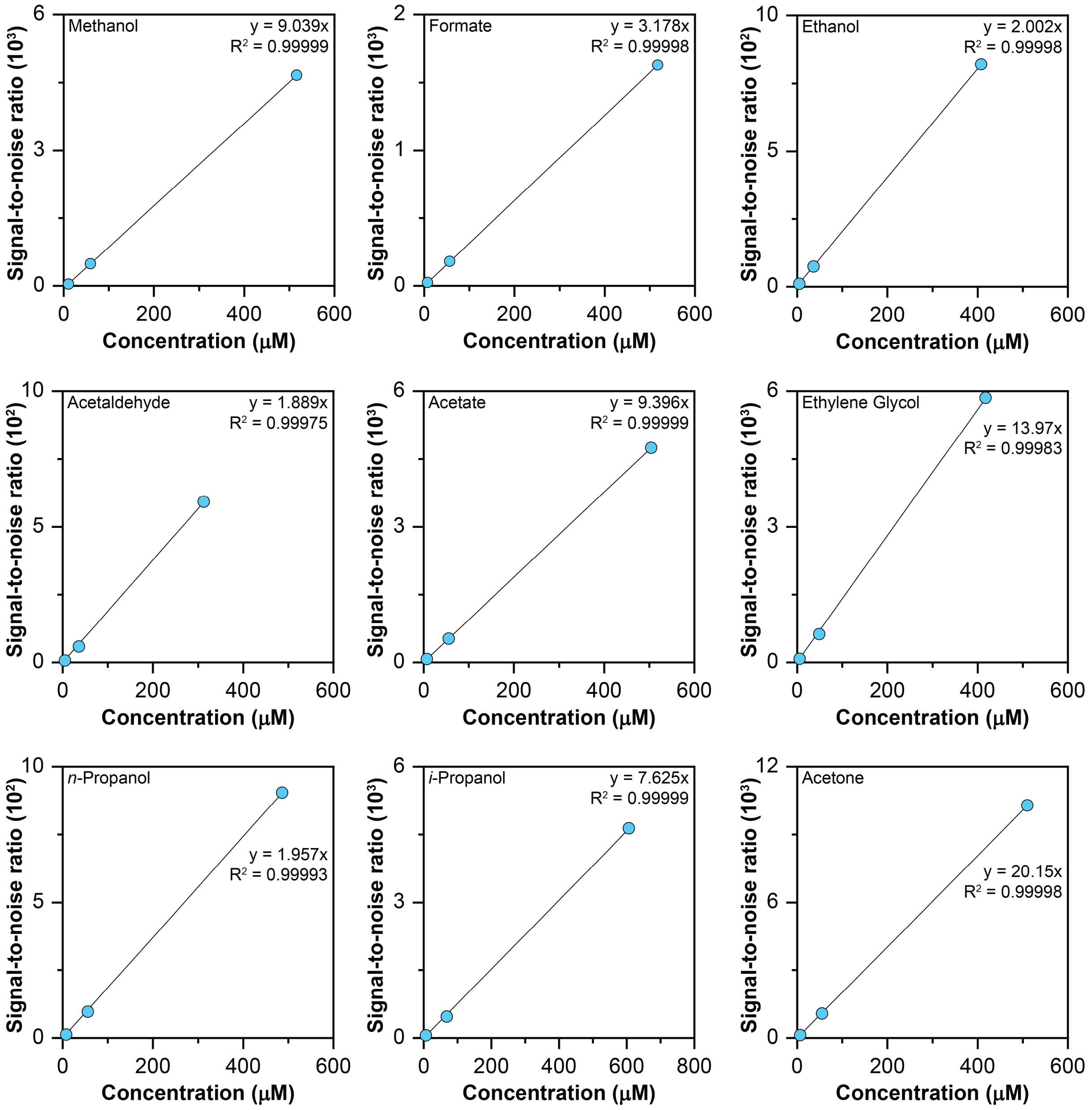

Supplementary Figure 6 | Determination of eCO_2RR product quantification limits on 500 MHz spectrometer. Signal-to-noise ratios as a function of concentration for compounds of reference sample are shown in **Supplementary Fig. 2**.

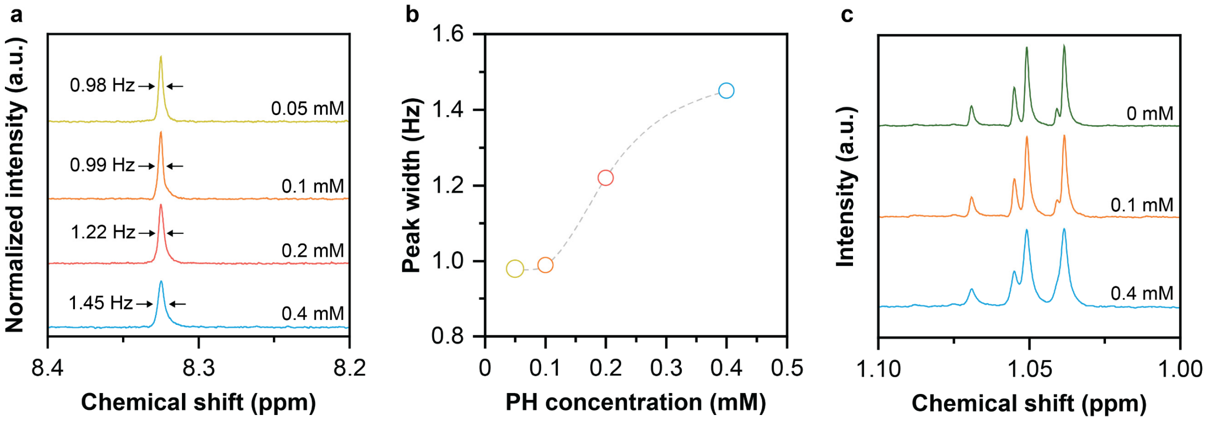

Supplementary Figure 7 | ProHance®-induced peak broadening. (**a**) Chemical shift region of formate of the reference sample shown in **Supplementary Fig. 2** after the addition of 0.05 (green), 0.1 (orange), 0.2 (red), and 0.4 mM ProHance® (blue). Spectra were recorded on a 300 MHz spectrometer. (**b**) Peak width as a function of ProHance® (PH) concentration. (**c**) Comparison of digital resolution within the chemical shift region of ethanol and *i*-propanol without (green) and with the addition of 0.1 (orange) and 0.4 mM ProHance® (blue). Spectra were recorded on a 500 MHz spectrometer.

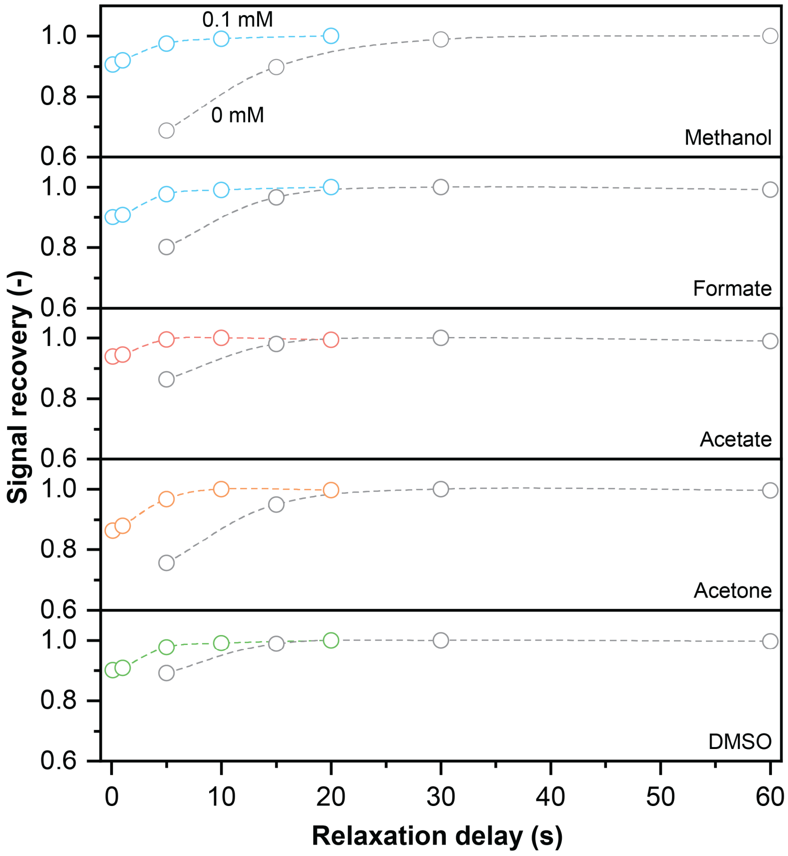

Supplementary Figure 8 | Influence of ProHance® addition on signal recovery. ^{1}H NMR spectra of the reference sample shown in **Supplementary Fig. 2** after the addition of 0.1 mM ProHance® were recorded with varying relaxation delays (d1) of 0.1, 1, 5, 10, and 20 s on a 500 MHz NMR spectrometer (64 scans). In comparison with the signal recovery values obtained without ProHance® from **Supplementary Fig. 3** in gray. Signal recovery values were calculated from peak areas at the respective chemical shifts provided in **Table 2**.

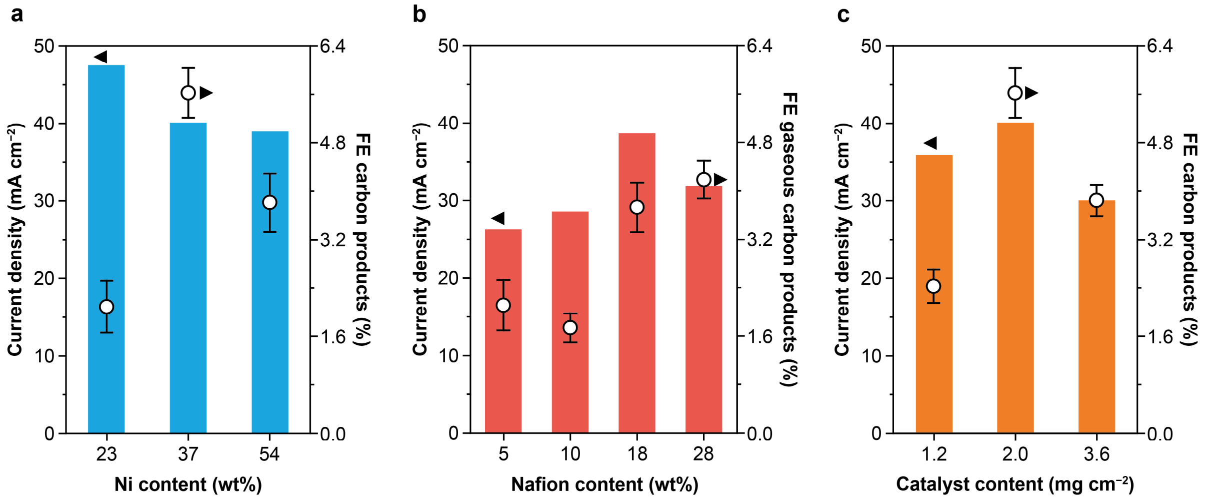

Supplementary Figure 9 | Current density and carbon product formation on PD-Ni electrodes with different architectures. Current densities and Faradaic efficiencies of carbon products formed over PD-Ni electrodes with varying (**a**) Ni, (**b**) Nafion™, and (**c**) catalyst contents. The configuration showing the better performance (as 37 wt% Ni, 18 wt% Nafion™, 2.0 mg cm^{-2} catalyst content) can be found in **c**. Experiments were performed at −1.0 V vs. RHE in 1 M $KHCO_3$. Error bars represent the standard deviation of three independent measurements.

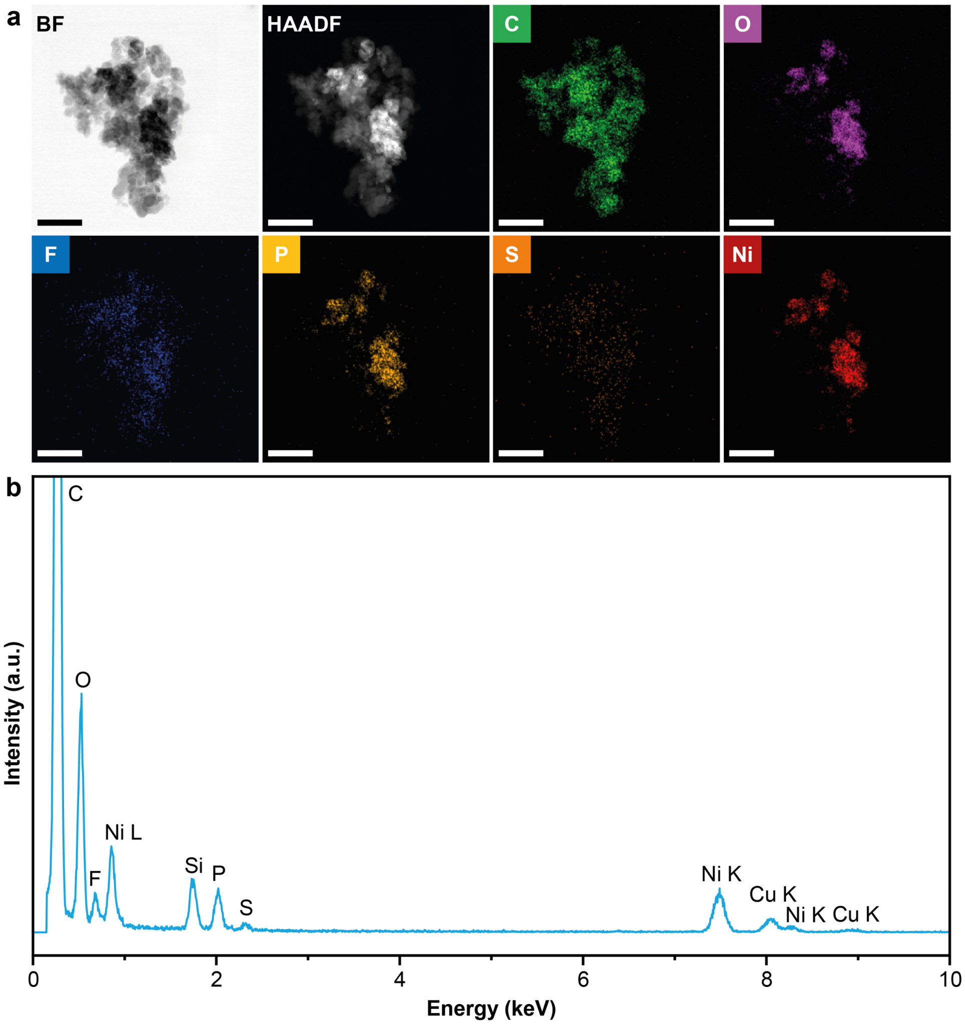

Supplementary Figure 10 | Single-element EDX maps and EDX spectrum of as-prepared Ni phosphate on carbon. (**a**) EDX maps of C K, O K, F K, P K, S K, and Ni K with corresponding BF- and HAADF-STEM images. (**b**) EDX spectrum from area investigated. F and S signals from Nafion™ ionomer, Cu signal from carbon-coated grid, and Si background signal from EDX detector system. Scale bars represent 200 nm.

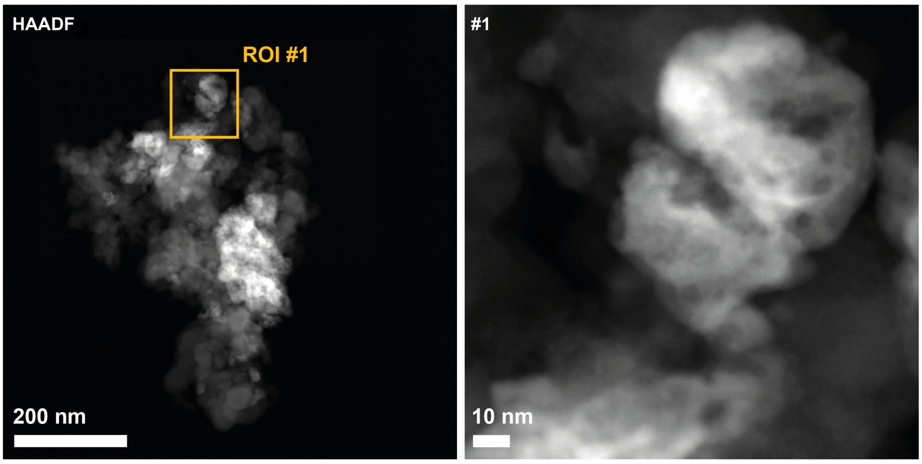

Supplementary Figure 11 | STEM imaging of as-prepared Ni phosphate on carbon. Overview HAADF-STEM image (left) with the indicated region of interest (ROI) and micrograph taken at high magnification (787k×) suggesting amorphous Ni phosphate phase.

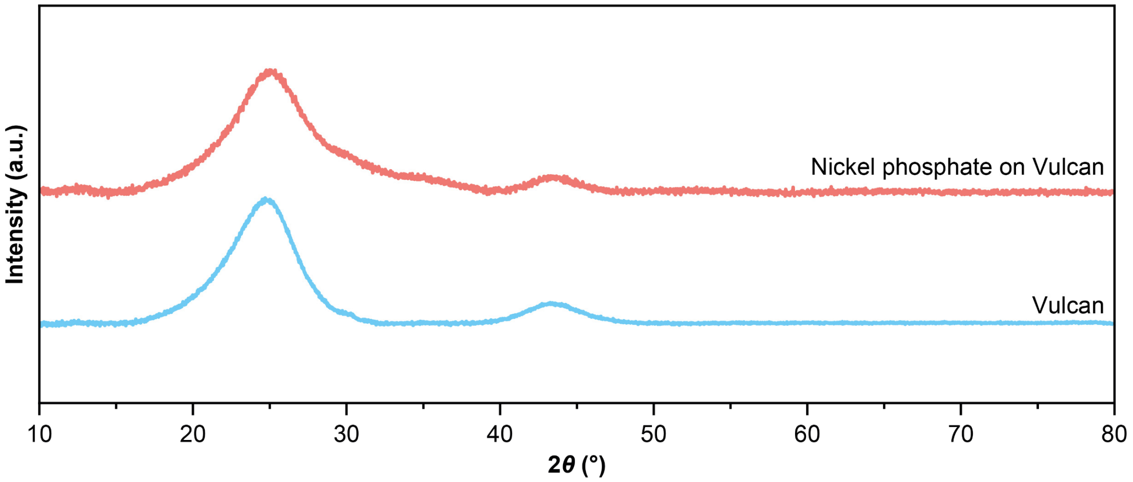

Supplementary Figure 12 | X-ray diffractogram of as-prepared Ni phosphate supported on carbon. XRD analysis of supported Ni phosphate (red) in comparison with bare Vulcan XC 72 support (blue). Diffractograms were baseline-corrected and normalized.

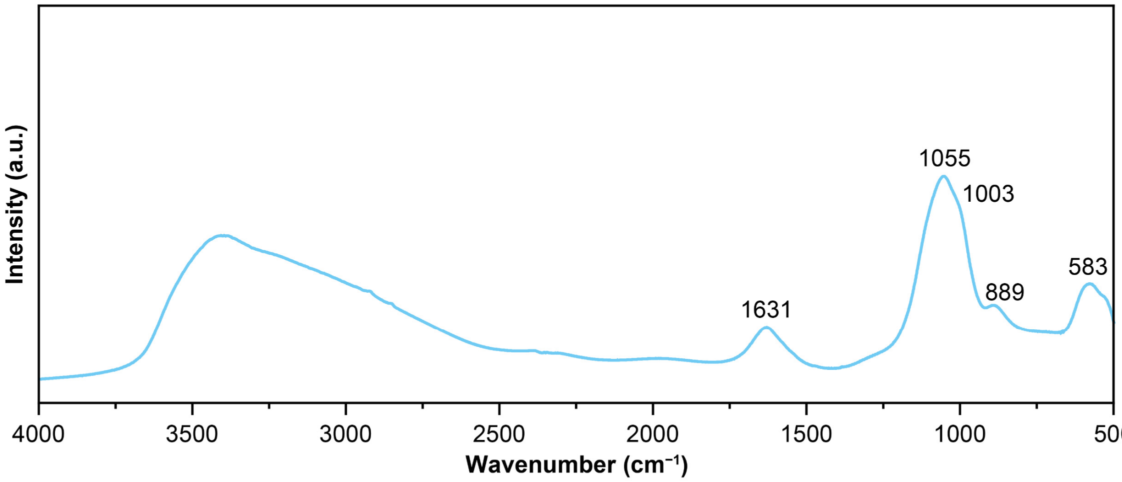

Supplementary Figure 13 | FTIR spectrum of as-prepared Ni phosphate. FTIR spectrum shows typical bands of Ni phosphates from P=O and P-O-P vibrations.[12] A clear assignment to Ni ortho- or pyrophosphate was not possible probably due to the overlapping of both phases.

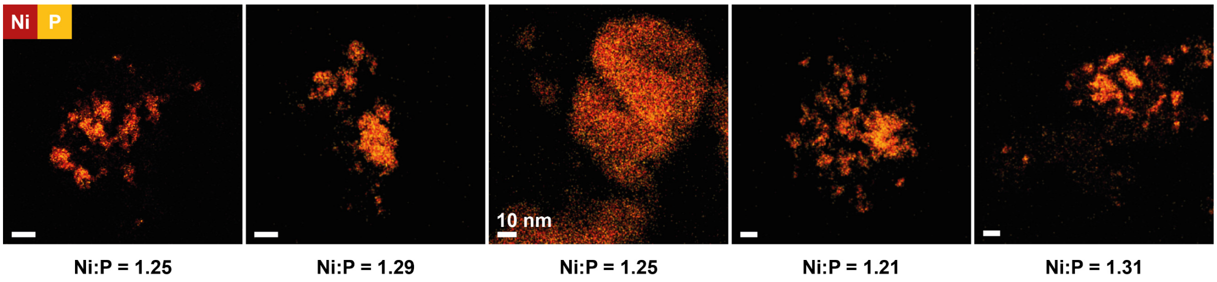

Supplementary Figure 14 | STEM area-selective analysis on as-prepared Ni phosphate on carbon. Superimposed EDX maps of Ni K and P K. Ni:P atomic ratios were obtained from corresponding EDX spectra. For representative single-element maps and EDX spectrum, see **Supplementary Fig. 10**. Scale bars represent 100 nm unless otherwise stated.

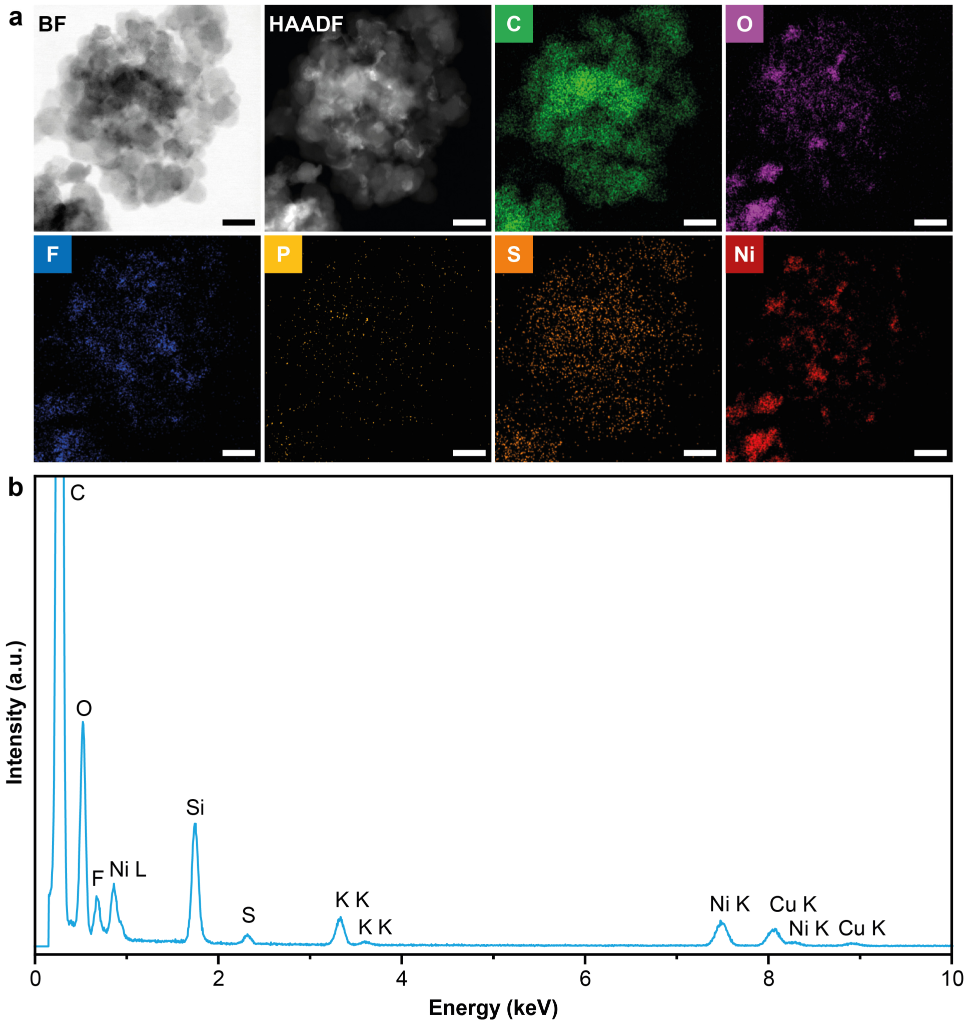

Supplementary Figure 15 | Single-element EDX maps and EDX spectrum of used PD-Ni catalyst. (**a**) EDX maps of C K, O K, F K, P K, S K, and Ni K with corresponding BF- and HAADF-STEM images. (**b**) EDX spectrum from area investigated. F and S signals from Nafion™ ionomer, K from residual $KHCO_3$ electrolyte, Cu signal from carbon-coated grid, and Si background signal from EDX detector system. Scale bars represent 50 nm.

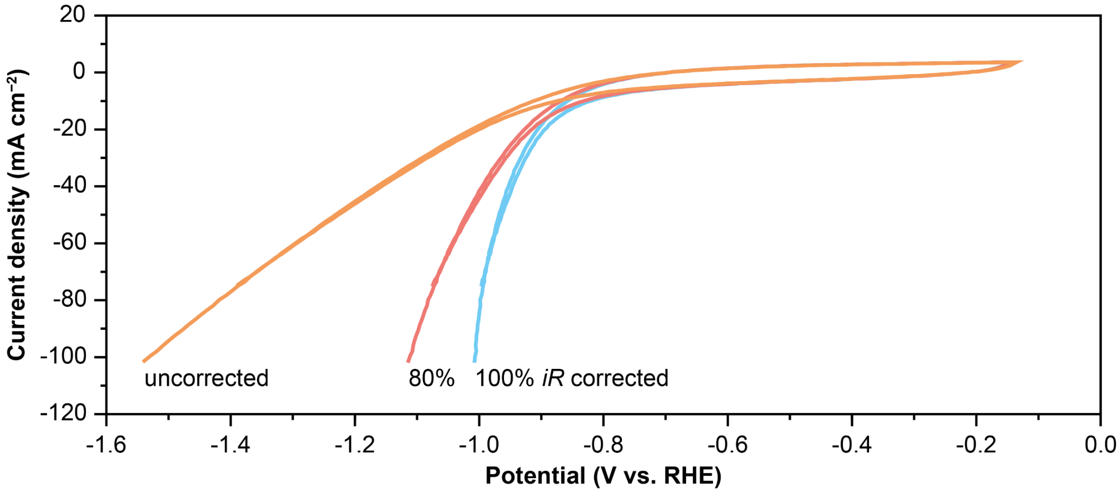

Supplementary Figure 16 | Cyclic voltammetry (CV) of PD-Ni catalyst. Uncorrected (orange) in comparison with 80% (red) and 100% post-corrected curves accounting for the *iR* drop. The *iR* drop compensation method used in study allows the compensation of 80% of the uncompensated resistance R_u in real-time without causing oscillations. To keep deviations between nominal and real potentials (determined by post-correction of the remaining 20% of R_u) low (<0.1 V), a potential range between −0.8 and −1.1 V vs. RHE were chosen. CV was performed in CO_2-saturated 1.0 M $KHCO_3$ (pH 7.8) with a scan rate of 100 mV s^{-1}.

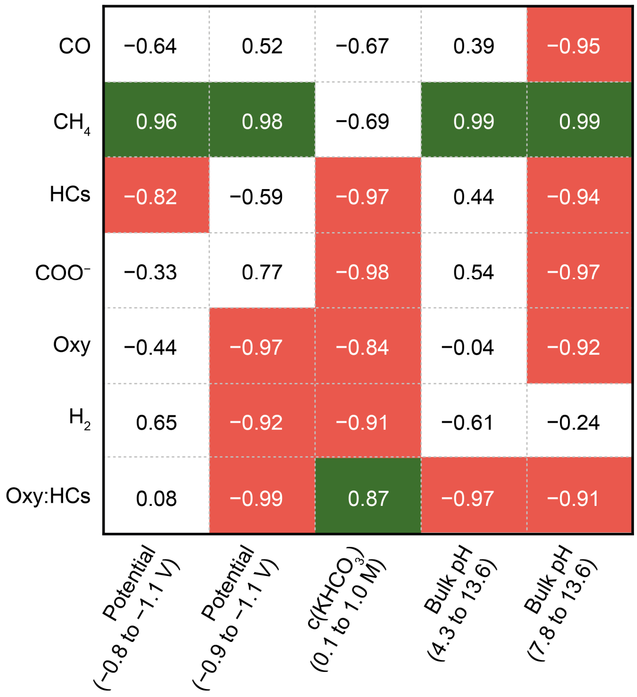

Supplementary Figure 17 | Pearson's correlation analysis of Faradaic efficiencies over PD-Ni catalyst. Colored areas indicate a significant positive (>0.8; green) and negative (<−0.8; red) correlation.

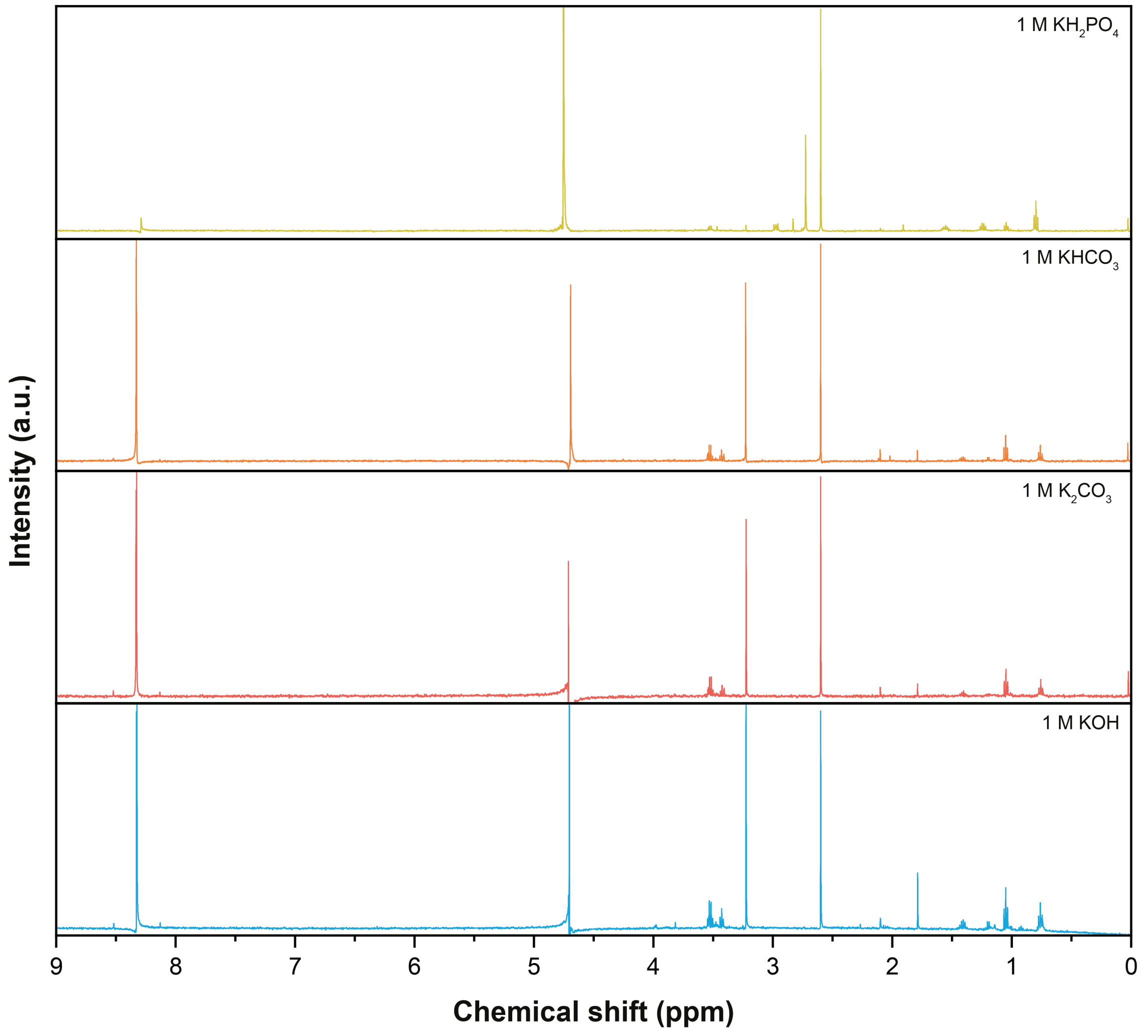

Supplementary Figure 18 | ^{1}H NMR spectra of catholytes after 184 min eCO_2RR. Spectra were recorded after eCO_2RR experiments in 1 M KH_2PO_4 (green), 1 M $KHCO_3$ (orange), 1 M K_2CO_3 (red), and 1 M KOH (blue) on a 500 MHz spectrometer.

Supplementary References

1. Nitopi, S., Bertheussen, E., Scott, S. B., Liu, X., Engstfeld, A. K., Horch, S., Seger, B., Stephens, I. E. L., Chan, K., Hahn, C., Nørskov, J. K., Jaramillo, T. F. & Chorkendorff, I. Progress and perspectives of electrochemical CO_2 reduction on copper in aqueous electrolyte. *Chem. Rev.* **119**, 7610–7672 (2019).
2. Sander, R. Compilation of Henry's law constants (version 4.0) for water as solvent. *Atmos. Chem. Phys.* **15**, 4399–4981 (2015).
3. Helmus, J. J. & Jaroniec, C. P. Nmrglue: an open source Python package for the analysis of multidimensional NMR data. *J. Biomol. NMR* **55**, 355–67 (2013).
4. Harris, C. R. *et al.* Array programming with NumPy. *Nature* **585**, 357–362 (2020).
5. Virtanen, P. *et al.* SciPy 1.0: fundamental algorithms for scientific computing in Python. *Nat. Methods* **17**, 261–272 (2020).
6. McKinney, W. *Proceedings of the 9th Python in science conference* **1**, 56–61 (2010).
7. Hunter, J. D. Matplotlib: A 2D graphics environment. *Comput. Sci. Eng.* **9**, 90–95 (2007).
8. Newville, M. *et al.* lmfit/lmfit-py: 1.2.1. (2023) doi:10.5281/ZENODO.7887568.
9. Pretsch, E., Bühlmann, P. & Affolter, C. *Structure determination of organic compounds*. (Springer Berlin, Heidelberg, 2000).
10. Rumble, J. *CRC handbook of chemistry and physics*. (CRC Press, Boca Raton, 2022).
11. Speight, J. G. *Lange's handbook of chemistry, seventeenth edition*. (McGraw-Hill Education, New York, 2017).
12. Theerthagiri, J., Cardoso, E. S. F., Fortunato, G. V., Casagrande, G. A., Senthilkumar, B., Madhavan, J. & Maia, G. Highly electroactive Ni pyrophosphate/Pt catalyst toward hydrogen evolution reaction. *ACS Appl. Mater. Interfaces* **11**, 4969–4982 (2019).
